# Naoxintong accelerates diabetic wound healing by attenuating inflammatory response

**DOI:** 10.1080/13880209.2021.1877735

**Published:** 2021-03-08

**Authors:** Leyu Fang, Lu Chen, Min Song, Juan He, Lusha Zhang, Chunxiao Li, Qianyi Wang, Wenjie Yang, Wei Sun, Yuze Leng, Hong Shi, Shaoxia Wang, Xiumei Gao, Hong Wang

**Affiliations:** a State Key Laboratory of Component-based Chinese Medicine, Tianjin, China; b Key Laboratory of Pharmacology of Traditional Chinese Medical Formulae, Ministry of Education, Tianjin University of Traditional Chinese Medicine, Tianjin, China; c Tianjin Key Laboratory of Chinese Medicine Pharmacology, Tianjin University of Traditional Chinese Medicine, Tianjin, China; d School of Integrative Medicine, Tianjin University of Traditional Chinese Medicine, Tianjin, China; e Institute of Traditional Chinese Medicine, Tianjin University of Traditional Chinese Medicine, Tianjin, China; f Buchang Pharmaceutical Co. Ltd., Xi'an, China

**Keywords:** Efferocytosis, macrophage polarization, ECM remoulding

## Abstract

**Context:**

Naoxintong (NXT), a prescribed traditional Chinese medicine, widely used in cerebrovascular and cardiovascular diseases, could be effective in diabetic wounds.

**Objective:**

This study evaluates the wound healing activity of NXT by employing an excisional wound splinting model.

**Materials and methods:**

NXT was dissolved in saline and given daily by gavage. Wounds were induced at the dorsum of non-diabetic (db/+) and diabetic (db/db) mice and treated with saline or 700 mg/kg/d NXT for 16 days. Wound closure was measured every four days. Extracellular matrix (ECM) remodelling, collagen deposition, leukocyte infiltration and expression of Col-3, CK14, CXCL1, CXCL2, MPO, Ly6G, CD68, CCR7, CD206, p-JAK1, p-STAT3 and p-STAT6 was analysed.

**Results:**

NXT significantly accelerated rate of wound closure increased from 70% to 84%, accompanied by up-regulation of collagen deposition and ECM at days 16 post-injury. Moreover, NXT alleviated neutrophil infiltration, accompanied by down-regulation of CXCL1 and CXCL2 mRNA expression. In addition, NXT markedly augmented neutrophil efferocytosis. In diabetic wounds, the levels of M1 marker gene (CCR7) increased, while M2 marker gene (CD206) decreased, demonstrating a pro-inflammatory shift. Application of NXT increased M2 macrophage phenotype in db/db mice. Mechanistically, NXT treatment increased expression level of p-STAT3 and p-STAT6 at days 3 post-injury, indicating NXT mediated macrophages towards M2 phenotype and alleviated inflammation in diabetic wounds by activation of STAT3 and STAT6.

**Conclusions:**

Our study provides evidence that NXT accelerates diabetic wound healing by attenuating inflammatory response, which provides an important basis for use of NXT in the treatment of chronic diabetic wound healing.

## Introduction

Wound healing is a major clinical problem in type 2 diabetes (T2D) and represents the leading cause of lower-extremity amputation in the world. Amputations are associated with substantial morbidity and a 3-year mortality rate of 50% (Terry et al. 2012). Therefore, development of alternative therapeutic approaches is urgent.

Wound healing encompasses a highly coordinated series of events requiring the interaction of various cell types to achieve tissue integrity and homeostasis after injury. To this end, wound repair proceeds in three overlapping but functionally distinct phases-initiating with an inflammatory phase marked by infiltration of neutrophils and macrophages, followed by a proliferative phase that includes tissue formation and extracellular matrix (ECM) deposition and concluding with a maturation phase that brings about matrix remodelling and resolution of the granulation tissue. Indeed, diabetic wounds are often associated with prolonged inflammation persistence. Inflammation begins immediately after wounding, neutrophils are the first immune cells to extravasate and infiltrate the wound, functioning to initiate debridement of devitalized tissues and to combat invading microbes (Bratton and Henson 2011). However, neutrophils must be eliminated in due course for wound healing to progress, as excessive or persistent neutrophil activity can cause further tissue damage and impede healing. Impaired clearance of apoptotic neutrophils is also associated with a variety of inflammatory diseases (Wood et al. 2014), and targeting excessive neutrophil activity has been proposed for treating diabetic wound injury (Jun et al. 2015).

Macrophages show marked phenotypic diversity and their secretory profile and functional properties are affected by micro-environmental factors. Classically activated (M1) and alternatively activated (M2) macrophages, which comprise the two principal macrophage populations, have been recognized. The former produce pro-inflammatory mediators, while the latter exhibit immune-regulatory properties. An imbalance between M1 and M2 macrophages occurs under physiological and pathological (allergic and chronic inflammation, tissue repair, infection and cancer) conditions *in vivo*. Prior studies in diabetic murine models have demonstrated that this critical early macrophage-mediated inflammatory response is impaired in diabetic wounds (Surboyo et al. 2020) which is partly due to macrophage dysfunction with an increased M1/M2 macrophage ratio (Yamashita et al. 2006; Vedadi et al. 2017).

Naoxintong (NXT), which contains 16 traditional Chinese medical herbs, has obtained the drug production approval in 1993. NXT is frequently applied for the treatment of cerebrovascular and cardiovascular diseases in clinic. In clinical application, it is also used for chronic complications of diabetes. NXT treatment before the onset of diabetes can target several diabetes related pathways, such as oxidative stress and inflammation, thereby preventing diabetes and its complications (Yang et al. 2019). However, the anti-inflammatory mechanism of NXT during diabetic wound repair is currently unknown. In this regard, it is noteworthy the role of NXT in the regulation of inflammatory response has been increasingly studied, which is closely related to its role in the macrophage polarization and neutrophil depletion (Wan et al. 2018; Yang et al. 2018). Previous studies found that NXT has resulted in a decreased M1/M2 macrophage ratio and shown that NXT plays an essential role in inhibiting neutrophil migration and injury amplification (Wang et al. 2017). Based on these, of particular interest to our efforts is that a possible role of NXT in inflammatory response induced by diabetic wounds is now emerging. In this study, we tested the hypothesis that NXT might attenuate inflammatory response induced by diabetic wounds.

## Materials and methods

### Animals

Adult male diabetic mice aged 8 weeks with blood glucose (30.14 ± 1.14 mM) and body weight (49.51 ± 3.88 g) and their control littermates with blood glucose (10 ± 4.13 mM) and body weight (25.38 ± 0.84 g) (db/db and db/+, respectively), were obtained from the Model Animal Research Center at Nanjing University (strain: BKS.Cg-Dock7^m+^/^+^Lepr^db/Nju^). The protocols for *in vivo* study with mice were approved by the Animal Ethics Committee of Tianjin University of Traditional Chinese Medicine (TUTCM20180611) and performed in accordance with the approved guidelines on the use of laboratory animals. Mice were housed in pathogen-free conditions at the Animal Center of Institute of Biomedical Engineering, Chinese Academy of Medical Sciences (Tianjin, China). They were maintained at controlled temperature (22–25 °C) and relative humidity (50–60%) on a 12 h light–dark cycle with free access to food and water and 3–5 mice per cage.

### Reagents

NXT (180524) was obtained from Shanxi Buchang Pharmaceutical Co. Ltd. (Xianyang, China). RIPA buffer (R0010) was purchased from Solarbio (Beijing, China). Avertin (T48402) was purchased from Sigma-Aldrich (St. Louis, MO). Protease inhibitor (0469132001), phosphatase inhibitor (0490837001), phenylmethylsulfonyl fluoride (PMSF, P0100), Transcriptor First Strand cDNA Synthesis Kit (4897030001) and FastStart Universal SYBR Green Master (4913914001) were bought from Roche (Mannheim, Germany). Trizol reagent (15596-026) was purchased from Life Technology (Waltham, MA). MPO protein was determined with an ELISA kit (Quantikine; R&D Systems, Minneapolis, MN). Other antibodies including CD68 (ab955), Ly6G (ab25377), CCR7 (ab32527), CD206 (ab64693), vimentin (ab45939), Col-3 (ab7778) and CK14 (ab7800) were provided by Abcam (Cambridge, UK).

### Surgical procedures and treatment

All the mice were anaesthetized by intraperitoneal injection of avertin (250 mg/kg). The excisional wound splinting model was generated according to the method described previously. After hair removal from the dorsal surface under anaesthesia, 6-mm full-thickness excision skin wounds were created on the midline of mice. A donut-shaped silicone splint was fixed around the wound and sewed up with 5-0 suture line. After treating gentamicin on the wounds, the wounds were covered with Tegaderm sterile transparent dressing to provide a waterproof, sterile barrier to external contaminants including liquids, bacteria and viruses and maintain a moist environment for wound healing. It was changed every two days. Then, 60 mice with skin wounds were divided into three groups: (1) sham group (*n* = 20). Non-diabetic mice (db/+) received saline solution orally. (2) Model group (*n* = 20). The db/db mice received saline solution orally. (3) NXT group (*n* = 20). The diabetic mice were orally administrated with 700 mg/kg/d NXT based on the dose used in clinical practice. NXT was dissolved in saline and given daily starting on day 0. NXT and saline solution were given for 16 consecutive days.

### Wound closure analysis

Wound closure was measured by tracing the wound area every four days using a camera. Wound closure was quantified by Image J software (Bethesda, MD) and wound healing was expressed as the percentage of the original wound area that had healed, calculated as [1 – (wound area day *x*/wound area day 0)] × 100%.

### Histological assessments

Wound skin tissues were fixed with 4% paraformaldehyde, embedded in paraffin and cut into sections (5 μm). They were incubated overnight at 60 °C and dehydrated with graded ethanol series for Masson’s trichrome staining. For immunohistochemical staining, the sections were covered with 3% H_2_O_2_ for 15 min at room temperature and antigen was retrieved by heat mediation for 15 min in a citrate buffer. After blocking with 5% bovine serum albumin (ALB) in Tris-buffered saline for 30 min at 37 °C, the sections were incubated with primary antibodies against (1:100 in PBS) at 4 °C overnight. Then, the slides were covered with biocatalytic secondary antibody (1:200 in PBS) for 30 min at 37 °C and streptavidin-horseradish peroxidase for another 15 min. Staining was visualized after incubation with a DAB–H_2_O_2_ solution. The slides were then counterstained with haematoxylin for 1 min, dehydrated with ethanol, and sealed in resinene for microscopic observation. For immunofluorescent labelling, sections were incubated with preheated antigen retrieval buffer for 15 min. Blocking was done by 5% bovine serum for 1 h at 37 °C. Primary antibody to anti-soybean agglutinin (1:100 in HBS) was incubated with the sections at 4 °C overnight. Secondary antibody DyLight^®^594 anti-goat IgG (H + L) (1:200 in HBS) was then incubated with the sections for 30 min at 37 °C. Finally, nuclei were stained with DAPI. The sections were viewed and photographed using a Nikon TI-U fluorescence microscope (Tokyo, Japan).

### Western blotting

Western blotting was performed to determine CCR7, CD206, Col-3, JAK1, p-JAK1/2, STAT1, p-STAT1, STAT3, p-STAT3, STAT6 and p-STAT6 protein expression at day 3 and day 7 post-injury. Wound tissues were homogenized in RIPA lysis buffer with a cocktail of protease and phosphatase inhibitors using tissue homogenizers. The mixture was spun 5000×*g* for 10 min to pellet unresolved fragments. Supernatants were collected and the protein concentration was measured by BCA method. After diluting in sample buffer [62.5 mM Tris–HCl, pH 6.8, 10% (v/v) glycerol, 2% SDS, 0.1% bromophenol blue], the protein samples were separated by 10% sodium dodecylsulphate polyacrylamide gel electrophoresis (SDS-PAGE) and transferred to PVDF membranes. Membranes were blocked with blocking buffer (5% skim milk or 5% BSA in TBS with 0.1% Tween 20) for 1 h. Membranes were then incubated with primary antibodies at 4 °C overnight. Eleven primary antibodies were used in the experiments: anti-CCR7 (ab32527), anti-CD206 (ab64693), anti-Col-3 (ab7778), anti-JAK1 (3344T), anti-p-JAK1/2 (66245S), anti-STAT1 (14994T), anti-p-STAT1 (9167S), anti-STAT-3 (9139T), anti-STAT3 (9145T), anti-STAT6 (5397S) and anti-p-STAT6 (56554S) primary antibodies. After washing, appropriate secondary antibodies were applied to the membranes for 1 h at room temperature and with relevant secondary antibodies goat anti-rabbit IgG (H + L) via standard techniques. The reactive bands were developed using chemiluminescence according to the manufacturer's instruction. Images were scanned and band intensities were analysed with Photoshop CS5 software (Adobe Systems, San Jose, CA).

### Enzyme-linked immunosorbent assay (ELISA)

To quantify MPO levels in the diabetic wound, wound samples were analysed using a mouse MPO ELISA (R&D Systems, Minneapolis, MN) according to the manufacturer’s instructions.

### Quantitative real-time reverse transcription polymerase chain reaction (qRT-PCR)

qRT-PCR analyses were performed via standard techniques. Briefly, total RNA was extracted with Trizol Reagent and reverse transcribed with a Transcriptor First Strand cDNA Synthesis Kit. Amplification was performed on QuantStudio6 Q6 real-time PCR systems (Applied Biosystems, Foster City, CA) by using FastStart Universal SYBR Green Master (Rox). Expression was calculated via the comparative-threshold cycle method and normalized to GAPDH or β-actin mRNA levels. The following primers were used: mouse C-X-C motif chemokine ligand 1 (CXCL1) (forward: ACTGCACCCAAACCGAAGTC; reverse: TGGGGACACCTTTTAGCATCTT), mouse C-X-C motif chemokine ligand 2 (CXCL2) (forward: ATCCAGAGCTTGAGTGTGACG; GTTAGCCTTGCCTTTGTTCAG), mouse GAPDH (forward: GCTACACTGAGGACCAGGTTGTC; AGCCGTATTCATTGTCATACCAGG).

### Assessment of renal and hepatic tissue injuries

The haematoxylin and eosin (H&E) staining was performed to detect renal and hepatic tissue morphology. Kidney function was evaluated via measurement of creatinine (CRE), and urea-nitrogen (BUN) concentration in the serum using autoanalyzer. The level of aspartate aminotransferase (AST), alanine aminotransferase (ALT), total bilirubin (TBIL), total protein (TP) and alkaline phosphatase (ALP) enzymes was also measured to evaluate hepatic injury.

### Statistical analysis

Statistical analysis was performed using SPSS software (version 16.0, Chicago, IL). The results from three independent experiments were expressed as mean ± SD. Differences between two groups were compared with Student's *t*-test. Values of *p* < 0.05 were considered to be statistically significant.

## Results

### NXT accelerates skin wound in diabetic mice

**Figure 1.**
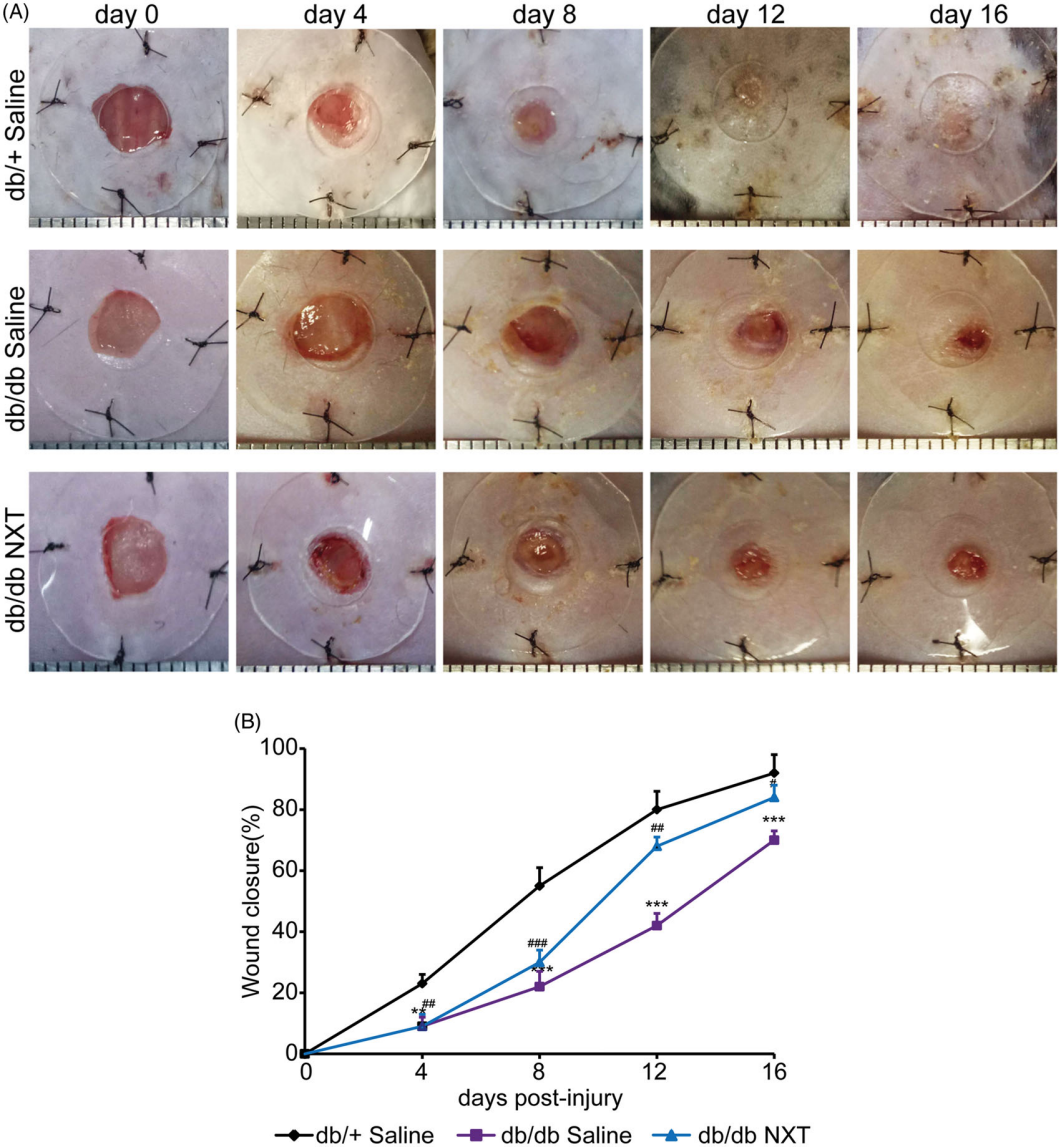
Effect of NXT on wound closure in db/db mice. (A) Representative photographs of wounds on day 0, 4, 8, 12 and 16. Scale bar = 1 mm. (B) The percentage of wound closure area at day 0–16 post-injury. Results were presented as mean ± SD. **p < 0.01 and ***p < 0.001 vs. db/+ mice treated with saline, ^#^*p* < 0.05, ^##^*p* < 0.01 and ^###^*p* < 0.001 vs. db/db mice treated with saline. *n* = 6.

In order to confirm whether NXT has an effect on chronic wound healing induced by diabetes, full-thickness excision wounds were created on the back of 8-week-old BKS. Cg-Dock7m+/+Leprdb/Nju (db/db) mice. These mice are leptin receptor deficient and represent a type II diabetes model characterized with obesity, hyperglycaemia and impaired wound healing. We routinely recorded body weight and fasting blood glucose in diabetic mice. There were no significant differences between saline group and NXT treated group, indicating NXT accelerates wound healing but not affects blood glucose within 16 days. The wound closure was examined every four days. The data reflected that the healing time in the db/+ control group was the shortest. The wound almost healed on day 12. The wound areas of the db/db mice were significantly increased than those in the db/+ mice from day 4 to day 16 (Figure 1(A,B); *p* < 0.05, *p* < 0.01 or *p* < 0.001, respectively). Thus, these data indicate that NXT is crucial for diabetic wound closure. In the Supplementary Material, we added NXT tissue toxicity *in vivo* test results. In this study, renal and hepatic functions were evaluated through measurement of CRE, BUN, AST, ALT, TBIL, TP, ALB and ALP in serum at 28 days after treatment with NXT at the concentration of 700 mg/kg/d (the experimental concentration was determined through clinical dosage). Histological changes in the liver and kidney tissues were observed by H&E staining at day 28 after treatment. Compared with saline group, no significant differences in either blood chemistry or histopathology were recorded. In NXT-treated mice, the hepatocytes are tightly arranged, the hepatic plate structure is clear, and there is no abnormality in the central vein and portal area. The renal cortex and medulla are clearly demarcated, the morphology of glomerular and renal tubular is normal in NXT-treated group. Our findings suggest that NXT treatment will not induce tissue toxicity in mouse liver and spleen.

### NXT enhances extracellular matrix deposition

**Figure 2.**
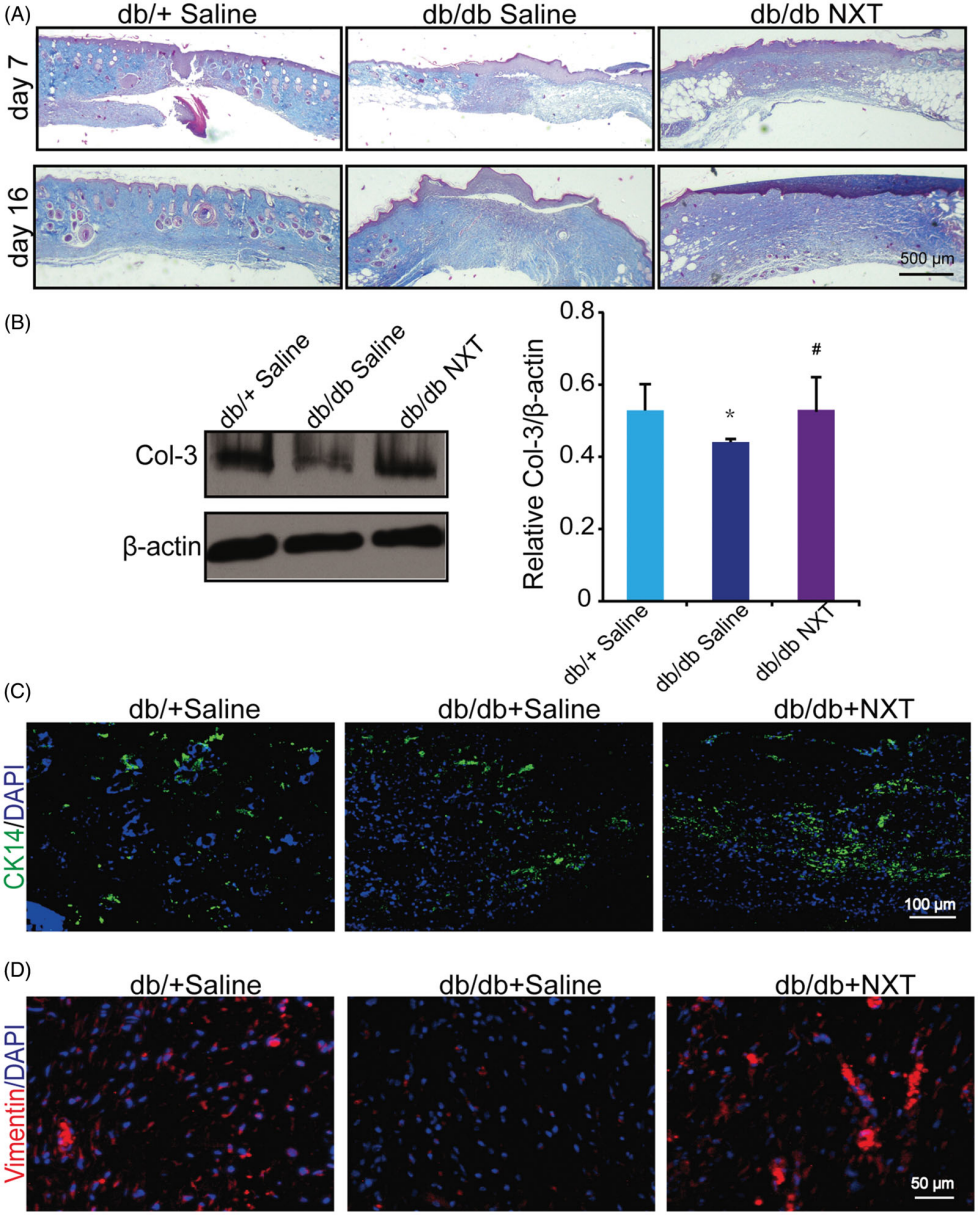
Effect of NXT on collagen deposition and ECM remoulding in diabetic wounds. (A) Masson’s trichrome staining wounds treated with NXT at day 7 and 16 post-injury. NXT increased collagen fibres in the wounds compared with saline. Collagen fibres were stained in blue. Scale bar = 500 μm. (B) Western blot of Col-3 and quantitative analysis of Col-3 at day 7 post-injury. (C) Immunofluorescence for CK14 at day 7 post-injury. Scale bar = 100 μm. (D) Immunofluorescence for vimentin at day 7 post-injury. Scale bar = 50 μm, and counterstained with DAPI. Results were presented as mean ± SD. *p < 0.05 vs. db/+ mice treated with saline, ^#^*p* < 0.05 vs. db/db mice treated with saline. *n* **=** 3.

To observe the effect of NXT on the production of new ECM in diabetic wounds, we analysed the deposition of local collagens using both Masson's trichrome and Western blotting. Total collagens were coloured blue by Masson's trichrome, and representative sections of NXT-treated wounds are shown in Figure 2(A). The collagens fibres were remarkably increased in NXT-treated diabetic mice when compared with untreated controls at day 16 post-injury. Meanwhile, Western blotting showed that the expression of ECM-associated proteins, such as collagen-3, was also significantly elevated in NXT-treated diabetic mice in compared with that of untreated controls (Figure 2(B)).

To further observe the ECM during tissue remodelling and regeneration during the course of wound healing. We than investigate the expression of several markers. We observed that NXT-treated diabetic wounds exhibited more staining of vimentin (red) and CK14 (green), at seven days than that of the controls (Figure 2(C,D)). Collectively, these findings indicate that NXT accelerates diabetic wound healing linked to up-regulation of ECM remoulding and collagen deposition.

### NXT promotes neutrophil efferocytosis during wound healing

Wound healing is usually impaired in clinical diabetes owing to dysregulated infiltration of leukocyte cells (Justet et al. 2015), and NXT has been involved in modulation of uncontrolled inflammatory responses by inhibiting neutrophil migration. Here, we investigated the therapeutic efficacy of NXT for diabetic wounds.

**Figure 3.**
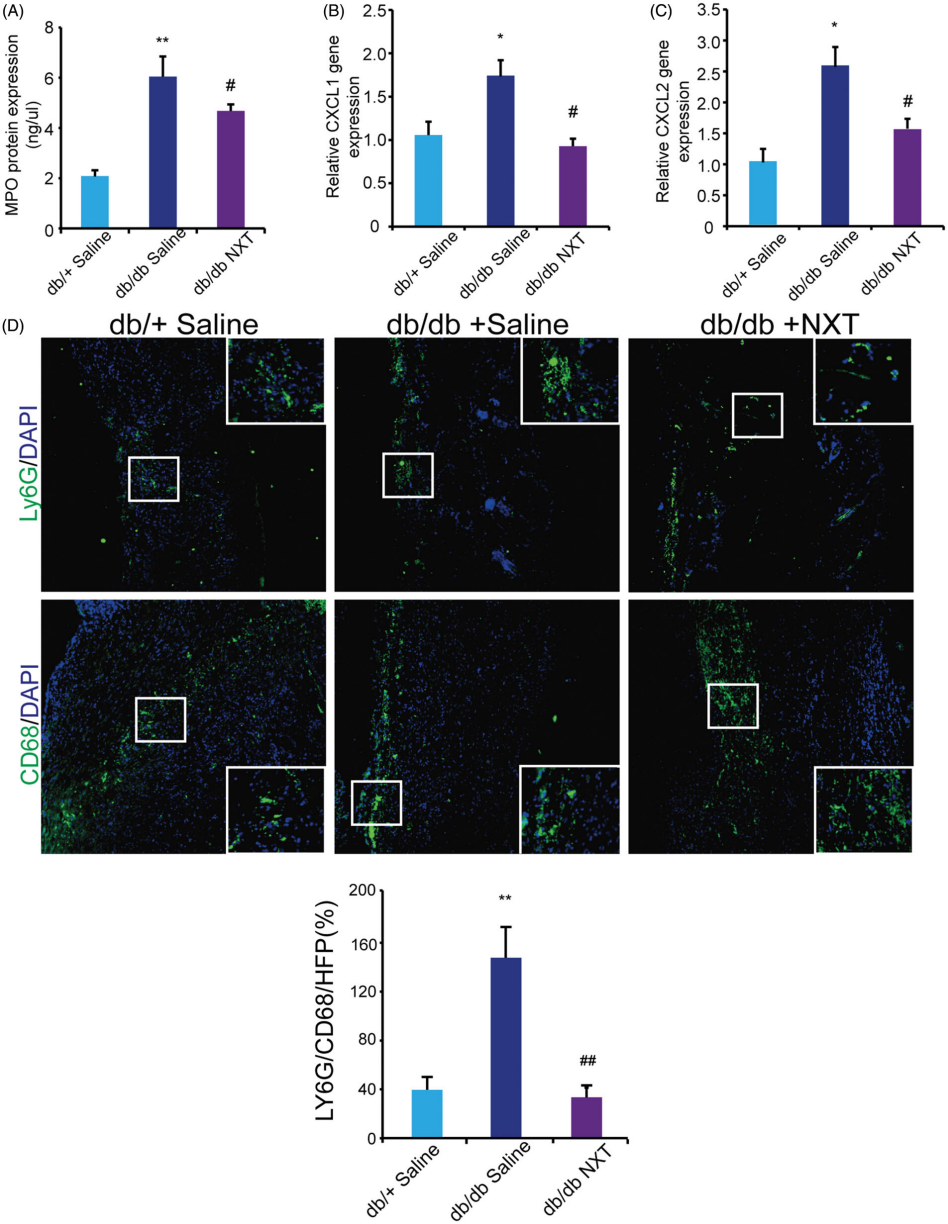
NXT decreased inflammatory response in diabetic wounds. (A) MPO protein expression at day 3 post-injury. (B, C) Relative gene expression of CXCL1 and CXCL2 at day 3 post-injury. (D) Immunofluorescence for Ly6G and CD68 at day 7 post-injury Scale bar = 100 μm. Results were presented as mean ± SD. *p < 0.05 and **p < 0.01 vs. db/+ mice treated with saline, ^#^*p* < 0.05 and ^##^*p* < 0.01 vs. db/db mice treated with saline. *n* = 3.

We analysed the expression of MPO protein to evaluate the extent of neutrophil infiltrates in diabetic wounds. The number of neutrophils significantly decreased at day 3 post-injury (Figure 3(A)). The increase in the number of neutrophils was attenuated by NXT. mRNA expression of the major neutrophil chemoattractants chemokine (C-X-C motif) ligand 1 (CXCL1) and CXCL2 significantly increased which was attenuated by NXT (Figure 3(B,C)). Macrophage phagocytosis of apoptotic neutrophils is vital for resolution of inflammation and tissue injury. Ly6G is a marker of neutrophils and CD68 is a marker of macrophages. Immunostaining for Ly6G and CD68 showed that neutrophils were accumulated in diabetic wounds. In NXT treated group, there is predominance of macrophages compared with the group receiving saline in db/db mice, but still a residual of neutrophils (Figure 3(D)). These results suggested NXT was able to induce apoptosis of neutrophils and efferocytosis of these cells by macrophages.

### NXT enhances the polarization of M2 macrophages through JAK/STAT pathway

**Figure 4.**
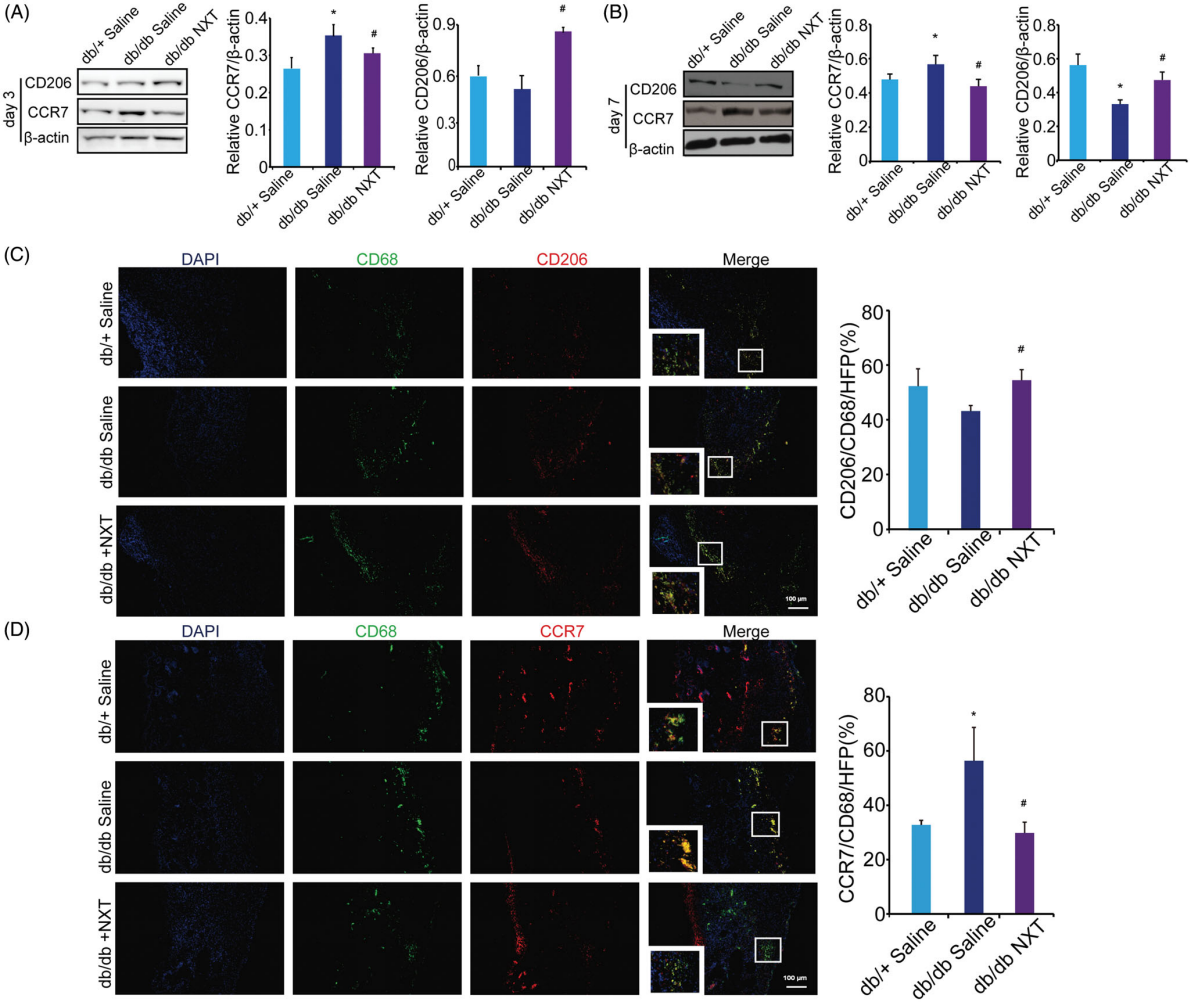
Effect of NXT on macrophage polarization in db/db mice. (A, B) Western blot of CD206 and CCR7 and quantitative analysis of CD206 and CCR7 in the wounds at day 3 and 7 post-injury. (C, D) Immunofluorescence for CCR7 and CD206 at day 7 post-injury. Scale bar = 100 μm. Results were presented as mean ± SD versus saline treated group, *p < 0.05 vs. db/+ mice treated with saline, ^#^*p* < 0.05 vs. db/db mice treated with saline. *n* = 3.

**Figure 5.**
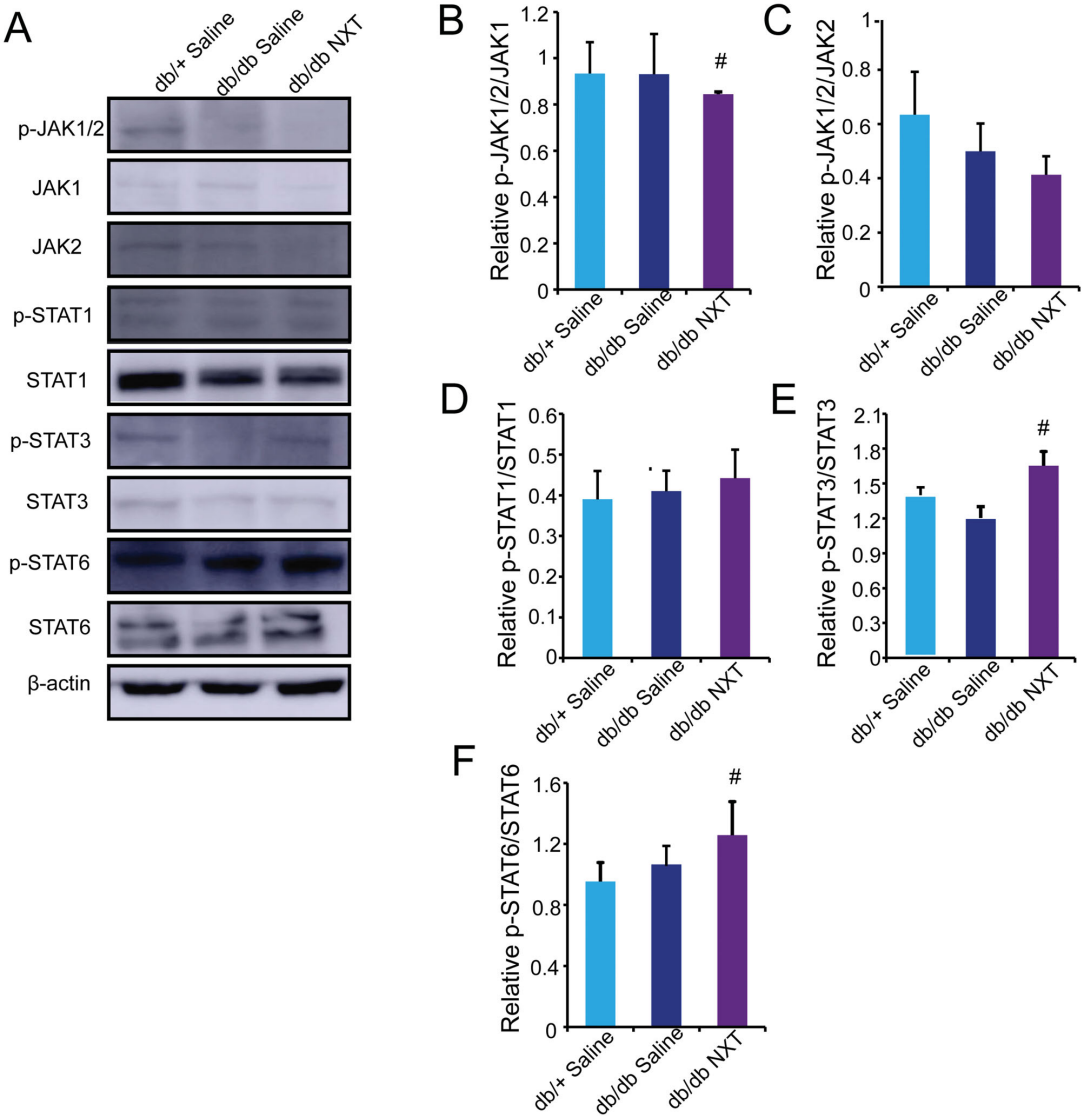
NXT regulated the JAK/STAT signalling pathway. (A–D) Western blot of p-JAK1/2, JAK1, JAK2, p-STAT1, STAT1, p-STAT3, STAT3, p-STAT6 and STAT6 and quantitative analysis of p-JAK1/2/JAK1, p-JAK1/2/JAK2, p-STAT1/STAT1, p-STAT3/STAT3 and p-STAT6/STAT6 in the wounds at day 3 post-injury. Results were presented as mean ± SD. ^#^*p* < 0.05 vs. db/db mice treated with saline, *n* = 3.

During the wound healing process, macrophages can adopt different phenotypes (M1 or M2) in response to environment stimuli. To assess the effects of NXT on macrophage polarization *in vivo*, we measured the protein level of M1 (CCR7) and M2 (CD206) at day 3 and 7 post-injury. As shown in Figure 4(A,B), Western blotting revealed that group receiving NXT reduced expression of CCR7 and increased CD206 compared to those receiving saline in db/db mice. We further examined the expression of M1 (CCR7) and M2 (CD206) markers by double immunofluorescent staining at day 7 post-injury, the expression of M2 markers peaked around day 7 post-injury whereas the expression of M1 markers peaked around day 3 post-injury, suggesting a progressive M2-to-M1 phenotype shift in both groups (Figure 4(C,D)). Accumulative evidence has shown that JAK/STAT-related signalling is a central pathway in controlling macrophage M1–M2 polarization. To investigate the underlying mechanism of the effect of NXT on JAK/STAT signalling pathway, we examined the effects of NXT on the inflammatory transcription factors JAK1, JAK2, STAT1, STAT3 and STAT6, which play crucial roles in macrophage polarization. The expression of p-JAK1/2, JAK1, JAK2, p-STAT1, STAT1, p-STAT3, STAT3, p-STAT6 and STAT6 was determined using Western blot analysis. As shown in Figure 5, NXT treatment significantly suppressed the JAK-1 phosphorylation and increased STAT3 and STAT6 phosphorylation at day 3 *in vivo*. However, p-STAT1/STAT1 expression was unaltered. Thus, these findings showed that JAK1, STAT3 and STAT6 pathways might participate in the macrophage polarization of NXT. *p < 0.05 vs. db/db saline ($\bar{x}\pm \text{SD},$ *n* = 3).

## Discussion

Restoration of tissue integrity and homeostasis following injury to the skin is of vital importance, as the integument provides the first barrier against invading microbes and pathogens. Acute inflammation occurring immediately after injury plays a critical role for host defence and debridement of necrotic tissues, although deregulated inflammation or its persistence can cause further tissue damage and lead to chronic non-healing wounds. Impaired wound healing causes considerable morbidity in afflicted patients, and is a leading cause of diabetes-related amputations. Thus, effective management of inflammation resolution is critical to wound care. Inflammatory response constitutes the first step in wound healing, and targeting excessive inflammation has been proposed for treating diabetic wounds.

This paper explores the potential role of NXT in a murine model of diabetic wound healing. Our results showed that wound healing is obviously accelerated in NXT-treated diabetic mice with wounds gaping narrower. Of note, NXT did not affect blood glucose within 16 days. Although the anti-diabetic effects of NXT have been observed in patients with hyperlipidaemia and hyperglycaemia, clinical trials suggest that targeting inflammatory cascade may differentially affect blood glucose over time at different concentration (Katsuhiro et al. 2018). Meantime, an enhancement in collagen deposition was also observed, implying an effect on the remould of ECM. Excessive neutrophils contribute to impaired healing, and thus wound closure in diabetic mice was accelerated by 50% on neutrophil depletion (McDaniel et al. 2013). We showed that NXT treatment leads to effective neutrophils removal in diabetic mice, consistent with curtailed inflammation. Moreover, an apparently increase M2-polarized macrophages was detected in wound sites from NXT-treated diabetic mice. Taken together, we have uncovered a critical role for NXT in triggering efferocytosis of neutrophils by macrophages and M2 polarization to promote the resolution of inflammation in diabetic wounds.

Marked neutrophil infiltration is a characteristic of the subacute phase response following diabetic injury. As resolution of acute inflammation is an active process that requires inhibition of further leukocyte recruitment and removal of leukocytes from inflamed sites, we evaluated the influence of NXT on MPO activity, which is an indicator of neutrophil migration and subsequent inflammation. The enzymatic and histological analyses reported herein demonstrated that diabetic injury markedly increased neutrophil infiltration and as well as the percentage of CD68 macrophages that contain Ly6G neutrophils. Efferocytosis, removal of cells undergoing apoptosis, is a key regulatory checkpoint in the inflammatory cascade for both the innate and adaptive immune systems (Van Vre et al. 2012). If this highly conserved process fails, secondary necrosis and then release of pro-inflammatory contents of neutrophils results; this leads to tissue damage (Felton et al. 2018). For example, pathological conditions such as acute respiratory distress syndrome (Hodge et al. 2011) are associated with delayed apoptosis and clearance of neutrophils, which worsens inflammatory injury. No further activation of macrophages is required for engulfment of apoptotic neutrophils and their ability to clear apoptotic cells is dependent on their programming to the M2 phenotype. This was evidenced by the finding that apoptotic Jurkat T cells released an unknown (lipid) factor that modulated NO production in peritoneal macrophages via Arg up-regulation (Johann et al. 2007). Our data show that NXT markedly enhances neutrophil depletion, which is mainly through the efferocytosis of neutrophils by macrophages in diabetic wound.

Macrophages are phenotypically highly plastic (Novak and Koh 2013), and their polarization state depends on the microenvironment present in the wounded area (Lucas et al. 2010). During the early stage, macrophages first exhibit the M1 phenotype to release TNF-α, IL-1β, IL-12 and IL-23 against the stimulus. But, if M1 phase continues, it can cause tissue damage. Therefore, M2 macrophages secrete high amounts of IL-10 and TGF-β to suppress the inflammation, contribute to tissue repair, remodelling, vasculogenesis and retain homeostasis (Sindrilaru et al. 2011; Gallagher et al. 2015). Notably, the pro-inflammatory-to-anti-inflammatory macrophage phenotype switch is impaired in multiple models of diabetes wherein a persistent hyper-inflammatory macrophage phenotype ensues (Leal et al. 2015). Therefore, the gradual switch in wound macrophage response from pro-inflammatory to anti-inflammatory is a key component of normal healing and is necessary for effective wound closure. In our study, macrophage phenotypes were confirmed by M1 (CCR7) or M2 (CD206) marker gene expression analysis. We found the increase in M1 marker gene expression and the decrease in M2 marker gene expression levels were remarkable in the inflammatory phase of diabetic. However, induction of NXT attenuated these alterations in protein levels, showing a shift in macrophage phenotype from M1 to M2. In addition, immunofluorescence analysis revealed consistent with results at the protein level. JAK/STAT signal pathway is involved in many important biological processes including cell proliferation, differentiation, apoptosis and immunoregulation (Abraham and Medzhitov 2011). STATs are widely found in cytoplasm, cell membrane and nuclei, which are a group of transcription factors that can be combined with the target gene promoter. STAT-3 and STAT-6 are the key transcription factor for the polarization of M2 macrophage. It combines with IRF4 and PPAR to activate the gene transcription process, then regulate the differentiation and maturation of M2 macrophage (Lee et al. 2019; Kung et al. 2020). Here, analysis of selected members of the JAK/STAT signalling pathway revealed the significant inhibition of JAK1 and activation of STAT3 and STAT6 phosphorylation, which are critical transcription factors for M2 activation. Our results showed that JAK1, STAT3 and STAT6 pathways might participate in the macrophage polarization of NXT.

In the clinical application, the treatment with a single drug might not achieve hoped-for efficacy in the treatment of diabetic wounds for the complexity of occurrence and development of the disease. NXT, with a specific combination of different herbs as formulae, provides a good treatment strategy as it takes full advantage of multiple effects of traditional medicine and synergistically deliver multiple bioactive effects and compromise potential toxicity. Previous studies have reported the potential effects of NXT against diabetes and certain complications. So far, more than 200 bioactive compounds in NXT have been identified. Among these molecules, tanshinone IIA, paeoniflorin (Dong et al. 2015), salvianolic acid B and hydroxysafflor yellow A (Hu et al. 2016; Bai et al. 2018) contained in NXT have been demonstrated biological functions on inflammatory response directly in both *in vivo* and *in vitro* studies. Collectively, the protection of NXT against diabetic wounds might be completed through its direct functions or indirect effects due to its anti-inflammatory activities. In the present study, the anti-inflammatory mechanism of NXT in diabetic wounds has been confirmed, which offers fresh perspectives and helps to provide better insight for the further diabetic application of NXT.

Considering the nature of NXT, a traditional medicine, we believe a few limitations, which are still needed to resolve by the further investigations. For instance, isolation and identification the major molecule(s) in NXT or its herbal medicine(s) are also important to understand the mechanisms by which NXT or its active component(s) reduces inflammation induced by diabetes.

## Conclusions

We found that NXT confers protection against diabetic wound by enhancing M2 macrophage polarization and efferocytosis. Activation of JAK/STAT signalling is a key for the M2 activation of NXT. Our results underscore a hitherto unrecognized role for NXT in diabetic injury, extending evidence for its anti-inflammatory properties in wound injury. Therefore, NXT may be a target for development of novel therapies for patients with diabetic injury.

## Supplementary Material

Supplemental Material
