# Supplementary material for: Naoxintong accelerates diabetic wound healing by attenuating inflammatory response
Source: Pharm Biol. 2021 Mar 8;59(1):250–9. doi: 10.1080/13880209.2021.1877735 (PMC7946048; doi:10.1080/13880209.2021.1877735)
Supplement: Supplemental Material [file IPHB_A_1877735_SM4821.zip › Supplementary Table 1.docx]

Table 1 Effects of NXT on hepatic and renal biochemical parameters in db/db mice at day 28.

| Group | ALT  (U/L) | AST  (U/L) | ALP  (U/L) | TBIL  (UM/L) | BUN  (mM/L) | CRE  (Μm/L) | TP  (U/L) | ALB  (U/L) |
| --- | --- | --- | --- | --- | --- | --- | --- | --- |
| Saline | 35.17±6.85 | 179.17±79.02 | 39.43±8.00 | 0.28±0.09 | 6.46±1.99 | 43.30±3.46 | 51.12±7.20 | 20.18±2.57 |
| NXT | 32.67±7.97 | 170.17  ±62.41 | 53.83  ±16.36 | 0.31  ±0.17 | 5.57  ±0.95 | 41.60±5.78 | 54.36  ±7.82 | 20.67  ±2.08 |

Renal and hepatic biochemical parameters were evaluated through measurement of creatinine (CRE), urea-nitrogen (BUN), aspartate aminotransferase (AST), alanine aminotransferase (ALT), total bilirubin (TBIL), total protein (TP), albumin (ALB), and alkaline phosphatase (ALP) in serum at 28 days after treatment with NXT at the concentration of 700 mg/kg/d. Values are represented as mean ± SE in group received saline and NXT. n = 6.
