# Supplementary figures and images for: Naoxintong accelerates diabetic wound healing by attenuating inflammatory response
Source: Pharm Biol. 2021 Mar 8;59(1):250–9. doi: 10.1080/13880209.2021.1877735 (PMC7946048; doi:10.1080/13880209.2021.1877735)

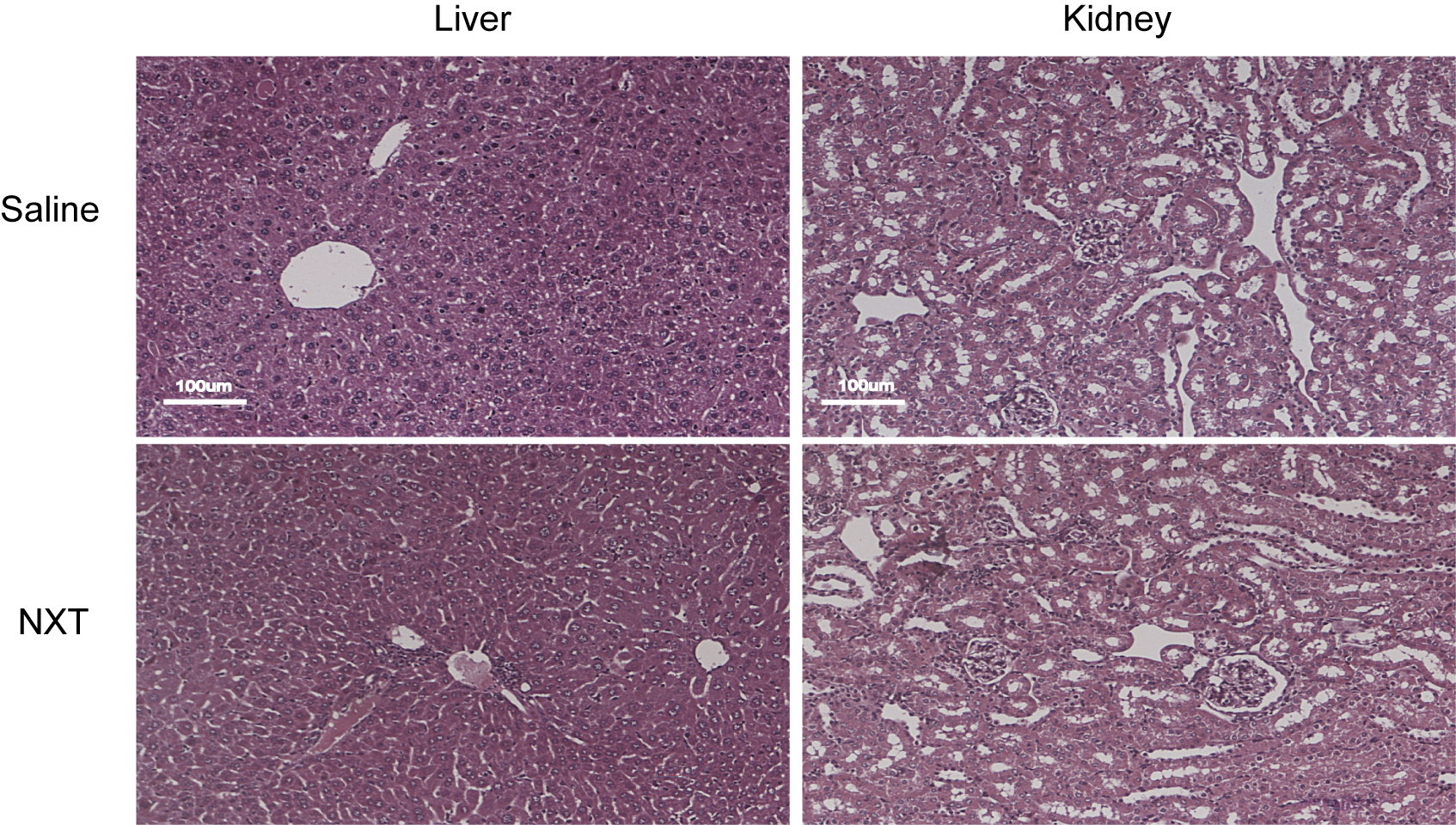

Supplement: Supplemental Material [file IPHB_A_1877735_SM4821.zip › Supplementary Figure 1.tif]
